# Introduction of Steam into the Practice of Dentistry

**Published:** 1849-04

**Authors:** 


### INTRODUCTION OF STEAM INTO THE PRACTICE OF DENTISTRY.

A FRIEND called in our office a few days since, and gave us the following: On his way up the river business took him across the river from Louisville, where he met an itinerating steam dentist. He was prepared with a small furnace and boiler. To the latter a flexible pipe was attached, which, when a patient presented himself with an aching tooth, or wished a nerve removed, was introduced into the cavity of the tooth; some *roots* or *herbs*, in flavor like garlic, were pu[illegible] into the boiler with water or some fluid. The furnace was the[illegible] heated up—puff, puff goes the steam, and with a hiss out jumps th[illegible] nerve—all done without pain, rights sold for the use of the invention on moderate terms, &c., &c. Our informant happened to have an aching tooth, which he put under treatment; but, unfortunately for the steam doctor, the nerve had long since departed, and the garlic vapo[illegible] could not restore it nor relieve the pain. Somewhat mortified, and feeling rather foolish, he *sloped.*—[CIN. ED.

